# An interdisciplinary view on dynamic models for plant genetics and morphogenesis: scope, examples and emerging research avenues

**DOI:** 10.3389/fpls.2013.00007

**Published:** 2013-01-31

**Authors:** Mariana Benítez

**Affiliations:** ^1^Departamento de Ecología de la Biodiversidad, Instituto de Ecología, Universidad Nacional Autónoma de MéxicoMexico City, Mexico; ^2^Centro de Ciencias de la Complejidad, Universidad Nacional Autónoma de MéxicoMexico City, Mexico

The iterative application of experimental and theoretical approaches to genetics and morphogenesis has proven extremely useful to many ends. On the one hand, alternating experimentation and modeling has allowed to integrate vast and complex sets of data into formal frameworks (Albert, 2007; Alvarez-Buylla et al., 2007; Pu and Brady, 2010). This alone has yielded a panoramic view of diverse study systems in plants, on which this editorial focuses, but also in animals, fungi, and prokaryotes (Alvarez-Buylla et al., 2008; Davidich and Bornholdt, 2008; Gonzalez-Rodriguez et al., 2012; Middleton et al., 2012), helping to identify openings and solving apparent inconsistencies in the sets of available data. On the other hand, mathematical models in genetics and morphogenesis have helped uncover some of the complex processes behind pattern and organ formation in plants, which has given rise to testable predictions that in turn input experimental work (Meinhardt et al., 1998; Espinosa-Soto et al., 2004; Prusinkiewicz et al., 2007; Azpeitia et al., 2010; Hamant et al., 2010; Mirabet et al., 2012). Yet, as a valuable and integrative approach, joint theoretical and experimental studies have opened new questions and research avenues in plant genetics, development and evolution, and thus face new challenges. Such challenges include assessing and comparing the scope and limitations of the diverse modeling frameworks and tools, as well as putting forward methods to study the emergence of spatiotemporal patterns from complex interactions among genes, proteins, hormones, physico-chemical mechanisms, environmental factors, among others.

The Research Topic *Dynamical Models in Plant Genetics and Morphogenesis*, jointly hosted by *Frontiers in Plant Genetics* and *Genomics and Plant Development and Evolution*, gathers contributions that represent different types of models in genetics and morphogenesis and critically discuss the scope of modeling methods. Strategies needed to establish a continuous feedback between experiments and theory are also discussed.

Some of the articles in this Research Topic focus on Boolean network models, a relatively simple type of dynamic model that has been successfully used to study different model systems. Greil (2012) reviews recent contributions to this field and discusses this tool as a modeling framework in plant genetics, exemplifying how the concept of *relevant components*—nodes that, due to the nature of their interactions with other nodes, have a key role in the overall network dynamics—can be used in the context of these models to simplify and further understand the systems under study. Azpeitia et al. (2011) suggest that Boolean network models can enhance traditional epistasis analyses and help design key experiments in molecular genetics.

This Research Topic also contributes to the identification of central achievements and unsolved issues in the field and discusses novel possible approaches. For example, although Boolean models rely on qualitative data and may not be sufficient to parametrize a continuous model, extensions of discrete models to continuous ones are important and instrumental, mainly because they enable the use of powerful analytical and numerical tools. Weinstein and Mendoza (2012) discuss a computational tool, SQUAD that takes Boolean models as a basis to build continuous dynamic models and further explore the rich dynamics of gene and biochemical regulatory networks. In turn, Carrillo et al. (2012) present an overview of the recently generated tools that enable the application of model checking, a technique for automatically verifying complex systems, such as biochemical networks.

The Research Topic also contains an exciting example of the application of dynamic modeling to the study of tissue growth in plants (Alim et al., 2012). Interestingly, this model considers a link between cell division and the mechanical stress to which cells are subjected, two central aspects of plant morphogenesis that are only starting to be coupled in dynamic models. Chitwood et al. (2012) point at the importance of morphometric analysis in the study of plant development and evolution, placing morphometry techniques and approaches as potential common ground for experimental and theoretical efforts.

Additionally, there are two contributions focusing on epistemic, conceptual and philosophic aspects of modeling, centering in plant developmental systems. Niklas and Kutschera (2012) analyze the approach consisting on studying metabolic and genomic subsystems belonging to a larger network of subsystems. This approach is exemplified by a broad review of two logic circuits and signal-activated subsystems that are central to plant development. The authors also illustrate the “subsystem incompleteness theorem,” which states that no subsystem is operationally self-sufficient, suggesting a whole-organism perspective to understand morphogenetic processes. Finally, Winther (2012) shows how Philosophy can shed light on mathematical modeling and on the juxtaposition of modeling and empirical data, discussing different functions of models and concluding that, while no single type of model can satisfy all the possible functions, valuable insights can arise from the explicitation of each model's functions and the integration of diverse models.

Overall, this panoramic picture of *Dynamical Models in Plant Genetics and Morphogenesis* has been delineated as an interdisciplinary and collective effort of researches and groups with different backgrounds, ranging from Molecular Genetics and Biophysics, to Philosophy of Science. Therefore, we expect that it will constitute a valuable representation of the field, at the same time it provides useful hints for future lines of research.
